# The Supraorbital Margin of Japanese Who Have No Visible Superior Palpebral Crease and Persistently Lift the Eyebrow in Primary Gaze is Higher and More Obtuse Than Those Who Do Not

**Published:** 2013-07-25

**Authors:** Yoshito Mishima, Kiyoshi Matsuo, Shunsuke Yuzuriha, Ai Kaneko

**Affiliations:** ^a^Department of Plastic and Reconstructive Surgery, Shinshu University School of Medicine, Matsumoto; ^b^Department of Plastic and Reconstructive Surgery, Hamamatsu University School of Medicine, Hamamatsu, Japan

## Abstract

**Objective:** Anthropological studies divided the Japanese into the Yayoi migrants, who had narrow eye, no visible superior palpebral crease, and high-positioned round supraorbital margins for cold tolerance, and the Jomon natives, who had wide eye, visible superior palpebral crease, and low-positioned straight supraorbital margins, thus suggesting an anatomical discrepancy between the vertical palpebral fissure and the height of the supraorbital margin. Because Japanese subjects without visible superior palpebral crease open their eyelids by lifting the eyebrows with the anterior lamella owing to increased tonic contraction of the frontalis muscle, we hypothesized that persistently lifting the eyebrows in primary gaze mechanically remodels the supraorbital margin to be high positioned and round. **Method:** We evaluated whether subjects without visible superior palpebral crease persistently lifted their eyelids more than subjects with visible superior palpebral crease, whether the presence of persistently lifted eyebrow in primary gaze affected the relative height of the supraorbital margin in coronal view 3-dimensional computed tomography imaging, and whether the shape of the supraorbital margin in the coronal view affected that in the sagittal view 3-dimensional computed tomography imaging. **Results:** Eyebrow height in subjects without visible superior palpebral crease was significantly larger than that in subjects with visible superior palpebral crease. The supraorbital margin of subjects without visible superior palpebral crease who persistently lifted the eyebrows in primary gaze was higher (rounder) and more obtuse than that of Japanese subjects with visible superior palpebral crease who did not. **Conclusions:** The mechanical pressure applied to the supraorbital margin by persistently lifting the eyebrows appears to functionally, rather than genetically, create the high (round) and obtuse supraorbital margin.

According to anthropological studies, the Japanese are genetically divided into the Neolithic Jomon natives and the Bronze Yayoi migrants from the cold Asian continent.^1^^-^6 The Yayoi migrants were reported to possess eyelid structures for cold tolerance, such as narrow eye, no visible superior palpebral crease (SPC), and high-positioned round supraorbital margin (Figs 1a, 2b, and 3a), whereas the Jomon natives were reported to have features of wide eye, visible SPC, and low-positioned straight supraorbital margin (Figs 1b, 2d, 2f, and 3b). However, there is an anatomical discrepancy between the vertical palpebral fissure and the height of the supraorbital margin; the narrow-eyed Yayoi migrants have higher-positioned round supraorbital margins, whereas the wide-eyed Jomon natives have lower-positioned straight supraorbital margins.

We have reported that the presence of a developed lower-positioned transverse ligament (LTL) between the superior-medial orbital rim and the lateral orbital rim on the lateral horn in the lower orbital fat space, around which the superficial levator aponeurosis turns upward to become the orbital septum, produces distinct features that include narrow eye, full eyelid, and no visible SPC, all of which correspond to characteristics of the Yayoi migrants (Fig 1a).^7^^-^9 To maintain an adequate visual field in primary gaze under these circumstances, increased contraction of the levator nonskeletal fast-twitch muscle fibers stretches the mechanoreceptors in Müller's muscle to enhance the levator skeletal slow-twitch muscle fibers and induce not only phasic, but also tonic, reflex contraction of the frontalis skeletal slow-twitch muscle fibers (Fig 1a).^8^^-^21

We have also reported that according to digital immobilization of eyebrow movement during eyelid opening and macro- and microscopic observation of the presence of visible SPC and the development of LTL, modern Japanese people are roughly divided into people without visible SPC who have a developed LTL and persistently lift the eyebrows (Figs 1a, 2b, and 3a) and people with visible SPC who have an undeveloped LTL and do not persistently lift the eyebrows (Figs 1b, 2d, 2f, and 3b).^9^ The former correspond to the Yayoi migrant ancestry, whereas the latter correspond to the Jomon native ancestry. Because a developed LTL antagonizes opening and folding of the anterior lamella of the upper eyelid in the Japanese without visible SPC, these individuals open their eyelids by lifting the eyebrow with the anterior lamella and the LTL owing to increased tonic contraction of the frontalis muscle, in addition to the retractile force of the levator aponeurotic expansions (Figs 1a, 2b, and 3a). In the Japanese with visible SPC, the undeveloped LTL does not antagonize opening and folding of the anterior lamella, and so they open their eyelids by folding the anterior lamella on the SPC via the retractile force of the levator aponeurotic expansions without the aid of lifting the eyebrow (Figs 1b, 2d, 2f, and 3b).

We hypothesized that since Japanese who have a developed LTL and no visible SPC always lift the eyebrows in primary gaze, their supraorbital margin may be high-positioned and round as a result of the lifting force by tonic contraction of the frontalis muscle, which mechanically presses on the supraorbital margin (Fig 1a). In contrast, because wide-eyed Japanese who have an undeveloped LTL and visible natural SPC do not lift the eyebrows on primary gaze, their supraorbital margin is presumed to be low-positioned and straight (Fig 1b).

To verify this hypothesis, we evaluated whether Japanese subjects who have no visible SPC and persistently lift the eyebrow in primary gaze had a high-positioned round supraorbital margin in comparison with those who do not.

## SUBJECTS AND METHODS

We enrolled 23 Japanese male subjects (24.5 ± 7.2 years old), who had sustained a fracture of the nose or orbital floor and who had been examined by 3-dimensional computed tomography (3D-CT) imaging of the orbit. The subjects did not have previous neurotoxin injected to their forehead or glabellar complex, previous upper lid blepharoplasty, or any eyelid ptosis or neuromuscular diseases. This cohort consisted of 10 subjects who had no visible SPC and who persistently lifted the eyebrows (Fig 3a) and 13 subjects who did not (Fig 3b). It was confirmed that digital immobilization of the eyebrow movement during eyelid opening restricted eyelid opening and folding in all subjects without visible SPC.

We evaluated whether the subjects without visible SPC persistently lifted their eyelids more than the subjects with visible SPC. Eyebrow height between the uppermost margin of the eyebrow above the center of the pupil and the intercanthal line were measured on the noninjured sides in primary gaze for statistical comparison of both group subjects without and with visible SPC. The measurements were based either on a 10-mm square scale (Casmatch, Dai Nippon Printing Co, Ltd, Tokyo, Japan) attached to the face or the corneal horizontal diameter measured after photographing.

Then, we evaluate whether the presence of persistently lifted eyebrow in primary gaze affected the relative height of the supraorbital margin in coronal view 3D-CT imaging. The vertical distance (VD) between the line of the horizontal distance (HD) and the highest point of the supraorbital margin was divided by the HD between the facial midline and the middle point of the frontozygomatic suture (Figs 3d, 3e). The resultant quotients (VD/HD) were compared between the groups.

Finally, we evaluated whether the shape of the supraorbital margin in the coronal view affected that in the sagittal view. The sagittal angles between a horizontal plane parallel to the Frankfurt plane and a plane tangent to the orbital roof at the highest supraorbital margin were measured in sagittal view 3D-CT images (Figs 4a-4d). A correlational analysis was performed between the above-determined VD/HD quotients in the coronal view and the sagittal view angles.

All subjects and patients gave informed consent to participate in the study, which was approved by our institutional review board for human subjects. Statistical analysis was performed using the Student *t* test and the Pearson correlation coefficient. A *P* value of less than .05 was used to indicate statistical significance.

## RESULTS

**Figure 2:**
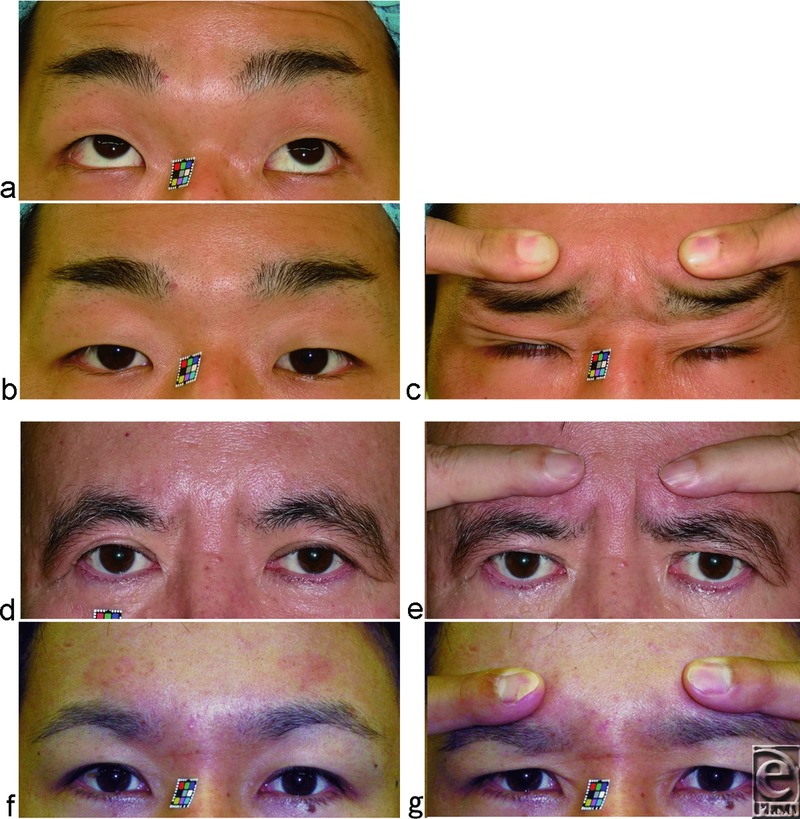
Digital immobilization of the eyebrow movement during eyelid opening classifies subjects as being without or with visible SPC as well as being with or without persistent lifting of the eyebrows in primary gaze. (*a*-*c*) A 25-year-old subject without visible SPC who did not have blepharoptosis but who persistently lifted the eyebrow in primary gaze. A 58-year-old man (*d*, *e*) and a 36-year-old man (*f*, *g*) with visible SPC who did not persistently lift the eyebrow in primary gaze.

**Figure 3:**
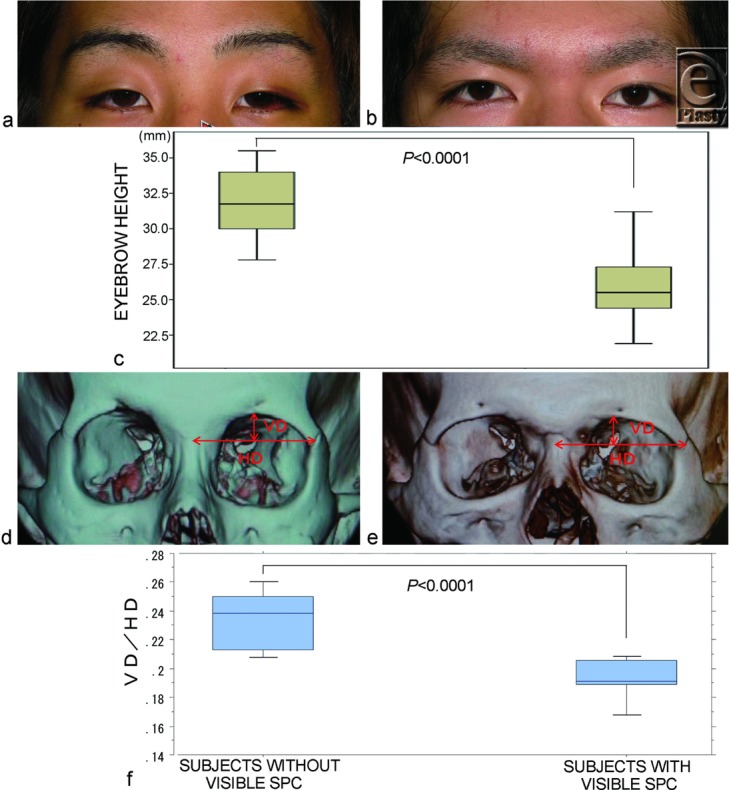
Differences in the eyebrow height in primary gaze and the coronal view of the supraorbital margin between subjects without and with visible SPC. (*a*) A representative 23-year-old subject without visible SPC who persistently lifted the eyebrows in primary gaze. (*b*) A representative 25-year-old subject with visible SPC who did not. (*c*) The eyebrow height in the subjects without visible SPC was significantly larger than that in the subjects with visible SPC. (*d*, *e*) The coronal views of 3D-CT imaging. To evaluate the relative height of the supraorbital margin, the vertical distance (VD) between the line of the horizontal distance (HD) and the highest point of the supraorbital margin was divided by the HD between the facial midline and the middle point of the frontozygomatic suture. (*f*) The relative height (VD/HD) in the subjects without visible SPC was significantly larger than that in the subjects with visible SPC.

**Figure 4:**
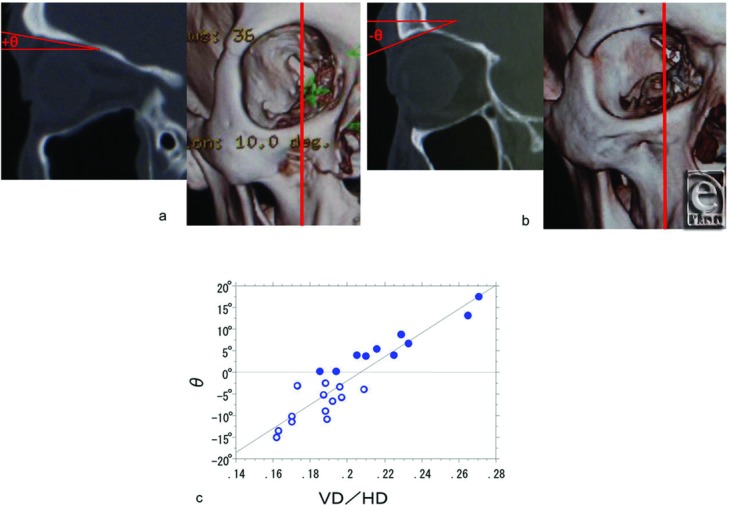
Correlation between the relative coronal height (VD/HD) of the supraorbital margin and the obtuseness of the sagittal angle (θ) of the supraorbital margin. The sagittal angles between a horizontal plane parallel to the Frankfurt plane and a plane tangent to the orbital roof were measured at the highest supraorbital margin in the sagittal view in 3D-CT images in a representative subject without visible SPC who persistently lifted the eyebrow in primary gaze (*a*) and a representative subject with visible SPC who did not (*b*). (*c*) The relationship between the relative coronal height (VD/HD) of the supraorbital margin and the obtuseness of the sagittal angle (θ) was significantly correlated (*r* = 0.925, *P* < .0001) for 10 subjects without visible SPC (filled circles) and 13 subjects with visible SPC (open circles).

Average eyebrow height in 10 subjects without visible SPC (31.86 ± 2.49 mm) was significantly larger than that in 13 subjects with visible SPC (25.94 ± 2.32 mm) (Fig 3c) (*P* < .0001). The VD/HD quotients of 10 subjects without visible SPC were significantly higher than those of 13 subjects with visible SPC (Fig 2f) (*P* < .0001). Furthermore, these quotients correlated significantly with the angles between the horizontal plane parallel to the Frankfurt plane and that tangent to the orbital roof at the highest supraorbital margin for all 23 subjects (Fig 3c) (*r* = 0.925, *P* < .0001).

## DISCUSSION

According to our evaluation of the coronal shape of the supraorbital margin using VD/HD, the supraorbital margin of 10 subjects without visible SPC who persistently lifted the eyebrow in primary gaze was significantly higher than that of 13 subjects with visible SPC who did not, even though the vertical palpebral fissure of the former appeared to be narrower. The relative height of the supraorbital margin appears not to be consistent with the vertical palpebral fissure, but rather with the height of the persistently lifted eyebrow. According to our analysis of the relationship between the coronal and sagittal shapes of the supraorbital margin, the relative coronal height significantly correlated to the obtuseness of the sagittal angle of the supraorbital margin among all subjects. These results suggest that the persistently lifted eyebrow due to the presence of tonic reflex contraction of the frontalis muscle in primary gaze may functionally lift the soft tissues around the supraorbital margin to mechanically apply pressure to the supraorbital margin, resulting in creation of characteristically high (round) and obtuse supraorbital margin in the subjects without visible SPC.

To explain the anatomical discrepancy between the vertical palpebral fissure and the relative height of the supraorbital margin of the 2 groups, Baba et al^4^ hypothesized that the facial flatness and narrow eye in Yayoi migrants for cold tolerance caused the brain to become larger and/or move anteriorly and enlargement of the maxillary sinus. Consequently, the face became extended in the vertical plane, resulting in a flat face. The supraorbital region shows a flat nasion and frontal sinus as well as a round supraorbital margin. However, it seems controversial that the round supraorbital margin, which more greatly exposes the orbital contents to the open air, is considered to be an adaptation to cold exposure, although since anthropological studies depended on skeletons and bones, they could not evaluate eyebrow movement due to tonic reflex contraction of the frontalis muscle as well as the presence of SPC.

The bones and articulations of the craniofacial skeleton grow and function in an environment of mechanical forces. These forces, which include muscle activity, mastication, the expansile growth of the brain, gravity, and man-made orthodontic appliances, influence the shape and relative position of each bone in the complex through the process of biological adaptation termed *remodeling*.^22^^-^24 For instance, orthodontic tooth movement is dependent on the remodeling of the periodontal ligament and alveolar bone by mechanical means,^24^^,^^25^ and the temporomandibular joint can be remodeled by Herbst treatment,^26^ wherein an increase in mandibular prognathism in both adolescents and young adults seems, in particular, to be a result of condylar and glenoid fossa remodeling. Furthermore, masseter muscle hypertrophy was reported to frequently accompany bone thickening in the region of the mandibular angle, even in unilateral cases^27^; the hyperostotic change was thought to be a secondary phenomenon to the mechanical stretching of the region of the mandibular angle by contraction of the masseter muscle. These reports suggest a possibility that the shape of the supraorbital margin may too be subject to remodeling by the mechanical force of the persistently lifted eyebrow by tonic contraction of the frontalis muscle in primary gaze.

Although we have proven an association between higher and more obtuse supraorbital margins in the group of subjects who had no visible SPC and persistently lifted the eyebrows, the cause of this relationship remains unclear and is only hypothesis driven. To prove causal relationship, Jomon Japanese or European patients with severe unilateral congenital ptosis, who persistently lift the eyebrow on the ptotic side and do not on the nonptotic side, should be evaluated. Because the small numbers of subjects were studied in the 2 groups to prove our hypothesis based on the anthropological discrepancy, a power analysis should be performed to determine the numbers required to prove or refute our hypothesis.

## CONCLUSIONS

The supraorbital margin of Japanese who have no visible SPC and persistently lift the eyebrow in primary gaze was higher and more obtuse than those who do not. The anatomical discrepancy between the vertical palpebral fissure and the height of the supraorbital margin appears to be solved. Because a developed LTL antagonizes opening and folding of the anterior lamella of the upper eyelid in Japanese subjects without visible SPC,^9^ these individuals open their eyelids by persistently lifting the eyebrow, which may create the high-positioned round supraorbital margin. In Japanese subjects with visible SPC, the undeveloped LTL does not antagonize opening and folding of the anterior lamella,^9^ and so they open their eyelids by folding the anterior lamella on the SPC without persistent lifting of the eyebrow, which may keep the supraorbital margin low-positioned and straight.

## Figures and Tables

**Figure 1:**
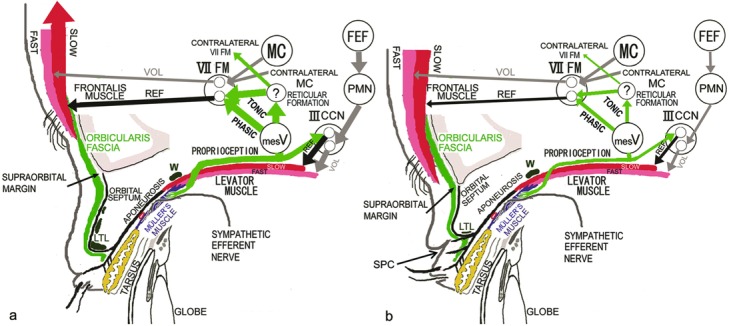
Anatomy and neurophysiology related to eyelid opening. (*a*) Subjects without visible superior palpebral crease (SPC) or Jomon natives. (*b*) Subjects with visible SPC or Yayoi migrants. ? indicates unknown nucleus; III CCN, central caudal nucleus of the oculomotor nuclear complex; FAST, fast-twitch muscle fibers; FEF, frontal eye field; LTL, lower-positioned transverse ligament; mesV, mesencephalic trigeminal nucleus; PHASIC, phasic contraction; PMN, premotor neurons in the rostral interstitial nucleus of the medial longitudinal fasciculus; REF, reflex contraction; SLOW, slow-twitch muscle fibers; TONIC, tonic contraction; VII FM, frontalis motor neurons; VOL, voluntary contraction; W, Whitnall's ligament.

## References

[1] Hanihara K (1991). Dual structure model for the population history of the Japanese. Japan Rev.

[2] Shinoda K, Kunisada T (1994). Analysis of ancient Japanese society through mitochondrial DNA sequencing. Int J Osteoarchaeol.

[3] Oota H, Saitou N, Matsushita T, Ueda S (1995). A genetic study of 2,000-year-old human remains from Japan using mitochondrial DNA sequences. Am J Phys Anthropol.

[4] Baba H, Narasaki A, Ohyama S (1998). Minatogawa hominid fossils and the evolution of late Pleistocene humans in east Asia. Anthoropol Sci.

[5] Oota N, Matsushita T, Ueda S (1999). Molecular genetic analysis of remains of a 2,000-year-old human population in China—and its relevance for the origin of the modern Japanese population. Am J Hum Genet.

[6] Ji X, Nakayama M, Han K, Liu X, Liu H, Kondo O (2005). Unique biological affinity of the hanging coffin people in ancient China based on craniometry of two skulls from Yunnan province. Anthropol Sci.

[7] Yuzuriha S, Matsuo K, Kushima H (2000). An anatomical structure which results in puffiness of the upper eyelid and a narrow palpebral fissure in the Mongoloid eye. Br J Plast Surg.

[8] Yuzuriha S, Matsuo K, Hirasawa C, Moriizumi T (2009). Refined distribution of myelinated trigeminal proprioceptive nerve fibres in Mueller's muscle as the mechanoreceptors to induce involuntary reflexive contraction of the levator and frontalis muscles. J Plast Reconstr Aesthet Surg.

[9] Ban M, Matsuo K, Ban R, Yuzuriha S, Kaneko A (2013). Developed lower-positioned transverse ligament restricts eyelid opening and folding and determines Japanese as being with or without visible superior palpebral crease. Eplasty.

[10] Sultana R, Matsuo K, Yuzuriha S, Kushima H (2000). Disinsertion of the levator aponeurosis from the tarsus in growing children. Plast Reconstr Surg.

[11] Matsuo K (2002). Stretching of the Mueller muscle results in involuntary contraction of the levator muscle. Ophthal Plast Reconstr Surg.

[12] Matsuo K (2003). Restoration of involuntary tonic contraction of the levator muscle in patients with aponeurotic blepharoptosis or Horner syndrome by aponeurotic advancement using the orbital septum. Scand J Plast Reconstr Surg Hand Surg.

[13] Yuzuriha S, Matsuo K, Ishigaki Y, Kikuchi N, Kawagishi K, Moriizumi T (2005). Efferent and afferent innervations of Mueller's muscle related to involuntary contraction of the levator muscle: important for avoiding injury during eyelid surgery. Br J Plast Surg.

[14] Kushima H, Matsuo K, Yuzuriha S, Kitazawa T, Moriizumi T (2005). The occipitofrontalis muscle is composed of two physiologically and anatomically different muscles separately affecting the positions of the eyebrow and hairline. Br J Plast Surg.

[15] Kondoh S, Matsuo K, Kikuchi N, Yuzuriha S (2006). Pathogenesis and surgical correction of involuntary contraction of the occipitofrontalis muscle that causes forehead wrinkles. Ann Plast Surg.

[16] Matsuo K, Yuzuriha S (2009). Frontalis suspension with fascia lata for severe congenital blepharoptosis using enhanced involuntary reflex contraction of the frontalis muscle. J Plast Reconstr Aesthet Surg.

[17] Ban R, Matsuo K, Osada Y, Ban M, Yuzuriha S (2010). Reflexive contraction of the levator palpebrae superioris muscle to involuntarily sustain the effective eyelid retraction through the transverse trigeminal proprioceptive nerve on the proximal Mueller's muscle: verification with evoked electromyography. J Plast Reconstr Aesthet Surg.

[18] Yuzuriha S, Matsuo K, Ban R, Yano S, Moriizumi T (2012). A contractile network of interstitial cells of Cajal in the supratarsal Mueller's smooth muscle fibers with sparse sympathetic innervation. Eplasty.

[19] Fujita K, Matsuo K, Yuzuriha S, Kawagishi K, Moriizumi T (2012). Cell bodies of the trigeminal proprioceptive neurons that transmit reflexive contraction of the levator muscle are located in the mesencephalic trigeminal nucleus in rats. J Plast Surg Hand Surg.

[20] Matsuo K, Osada, Ban R (2013). Electrical stimulation to the trigeminal proprioceptive fibers that innervate the mechanoreceptors in Müller's muscle induces involuntary reflex contraction of the frontalis muscles. J Plast Surg Hand Surg.

[21] Matsuo K, Ban R (2013). Surgical desensitization of the mechanoreceptors in Müller's muscle relieves chronic tension-type headache caused by tonic reflexive contraction of the occipitofrontalis muscle in patients with aponeurotic blepharoptosis. J Plast Surg Hand Surg.

[22] Moffett B (1971). Remodelling of the craniofacial articulations by various orthodontic appliances in rhesus monkeys. Trans Eur Orthod Soc.

[23] Moffett BC, Zingeser MR (1973). Remodelling of the craniofacial skeleton produced by orthodontic forces. Symposia of the Fourth International Congress of Primatology.

[24] Meikle MC (2007). Remodeling the dentofacial skeleton: the biological basis of orthodontics and dentofacial orthopedics. J Dent Res.

[25] Meikle MC (2006). The tissue, cellular, and molecular regulation of orthodontic tooth movement: 100 years after Carl Sandstedt. Eur J Orthod.

[26] Ruf S, Pancherz H (1999). Temporomandibular joint remodeling in adolescents and young adults during Herbst treatment: a prospective longitudinal magnetic resonance imaging and cephalometric radiographic investigation. Am J Orthod Dentofacial Orthop.

[27] Beckers HL (1977). Masseter muscle hypertrophy and its intraoral surgical correction. J Maxillofac Surg.

